# Supplementary material for: Reversible histone glycation is associated with disease-related changes in chromatin architecture
Source: Nat Commun. 2019 Mar 20;10:1289. doi: 10.1038/s41467-019-09192-z (PMC6426841; doi:10.1038/s41467-019-09192-z)
Supplement: Supplementary file 1 — Supplementary Information [file 41467_2019_9192_MOESM1_ESM.pdf]

Reversible Histone Glycation is Associated with Disease-Related Changes in Chromatin Architecture

David et al.

Supporting Information Table of Contents

Supplementary Figure 1. MGO glycation of full length H3...3

Supplementary Figure 2. Persistent histone glycation throughout the cell cycle......................4

Supplementary Figure 3. Analysis of NCP components on a native gel western blot...............5

Supplementary Figure 4. ATAC-seq analysis...6

Supplementary Figure 5 Biotinylated H3 peptide synthesis..7

Supplementary Figure 6. Recombinant expression of wild type and C106A mutant DJ-1........8

Supplementary Figure 7. NCPs glycation and deglycation...9

Supplementary Figure 8. Overexpression of DJ-1 in MGO treated 293T cells.......................10

Supplementary Figure 9. Cell viability..11

Supplementary Figure 10. DJ-1 and histone PTMs levels..12

Supplementary Figure 11. DJ-1 expression in breast cancer cell lines and tumors................13

Supplementary Figure 12. SKBR3 show lower viability after DJ-1 Knockdown.....................14

Supplementary Figure 13. Proposed mechanism of DJ-1-mediated histone deglycation......15

Supplementary Figure 14. Full gels and uncropped immunoblots ……...............................16

Supplementary Table 1. Primary antibodies used in this manuscript....................................17

Supplementary Table 2. Secondary antibodies used in this manuscript................................18

Supplementary Table 3. MGO treatments used in this study...19

Supplementary Figure 1. MGO glycation of full length H3. Recombinant H3 was incubated with the indicated concentrations of MGO, after which the samples were separated on SDS-PAGE and analyzed by western blot with the indicated antibodies.

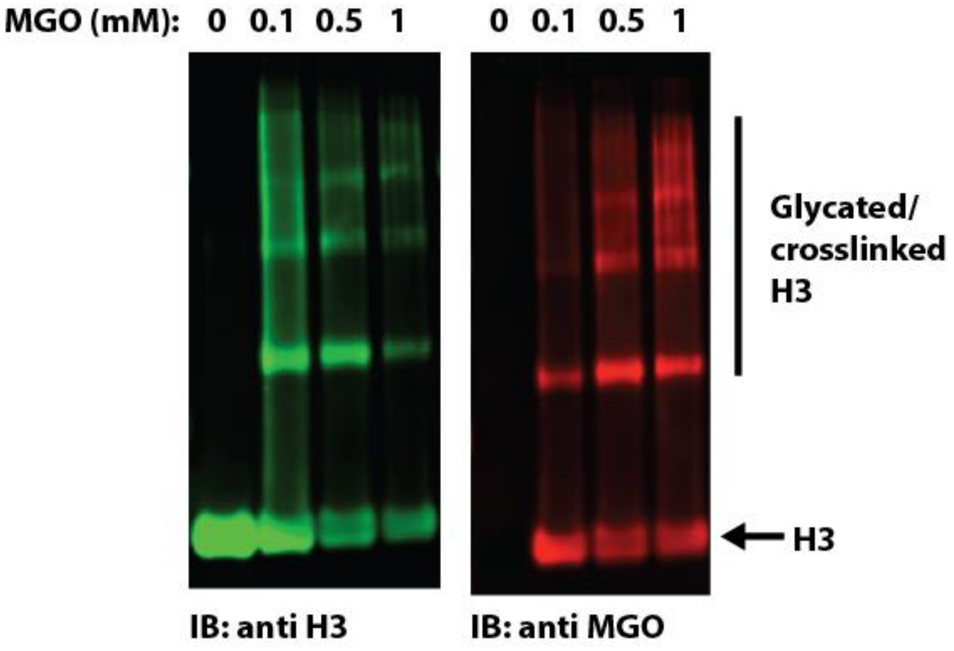

Supplementary Figure 2. Persistent histone glycation throughout the cell cycle. 293T cells were pulsed for 36 or 12 hours with 1 mM MGO followed by an MGO-free media chase of 0, 12 or 24 hours. Cells were harvested and fractionated to a soluble protein and chromatin-bound histones fractions. Equal total protein sample was loaded on an SDS-PAGE followed by a western blot analysis with the indicated antibodies.

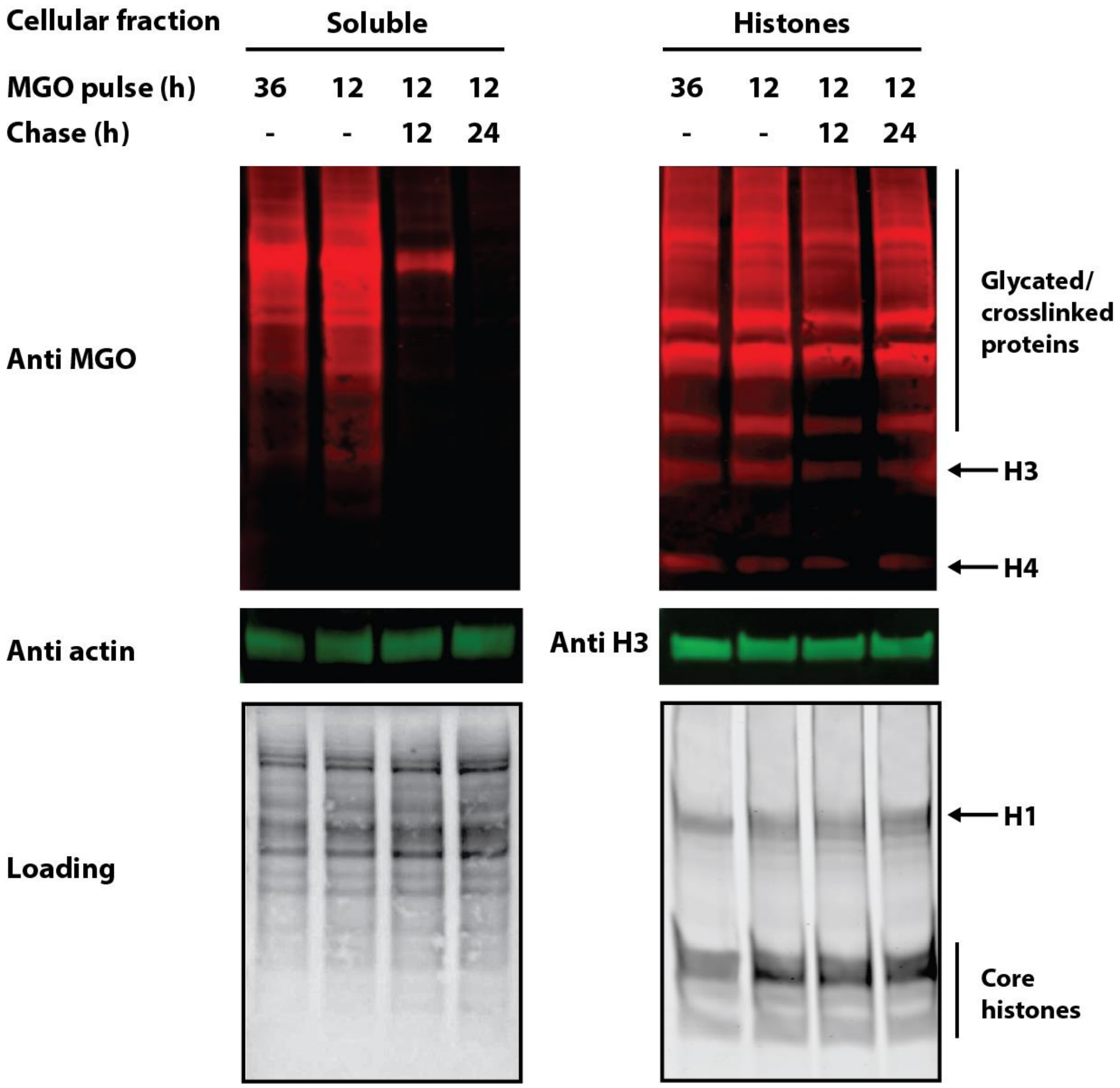

Supplementary Figure 3. Analysis of NCP components on a native gel western blot. The isolated components of the NCPs (601 DNA and H3 as a representative histone) as well as assembled NCPs contining eithere non-biotinylated or a biotinylated 601 were separated on a native gel and either stqaned with EtBr to visualize DNA or transferred to a membrane and blotted with either anti H3 or anti biotin. The results show that free histones or DNA do not appear on a tranferred native gel.

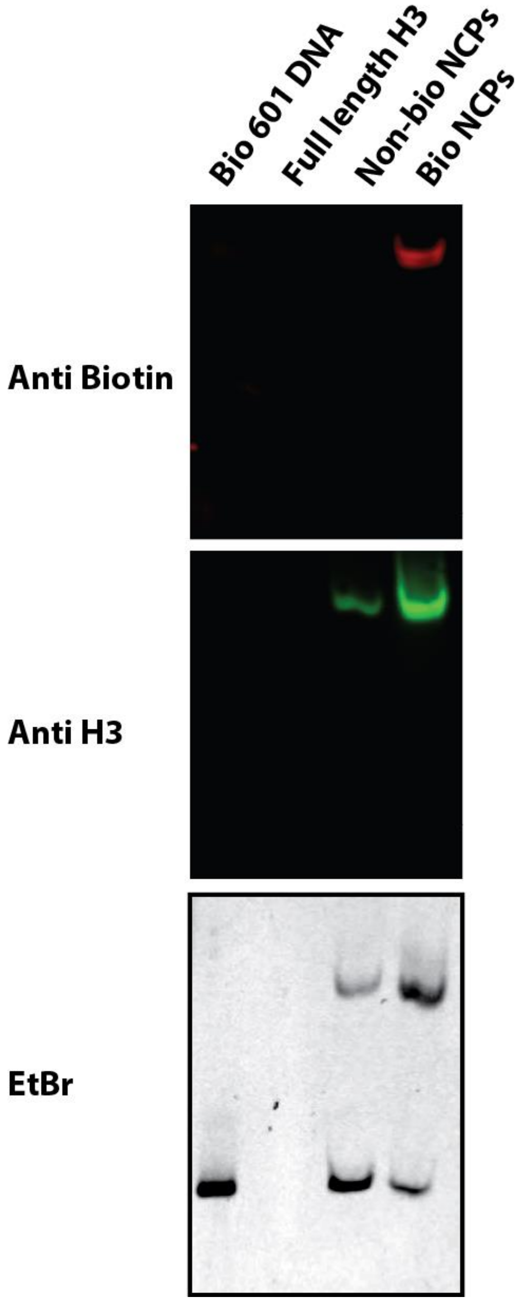

Supplementary Figure 4. ATAC-seq analysis. The distribution of ATAC-seq library insert size show stereotypical periodicity that represent enrichment of nucleosome free regions (< 149bp), mono-nucleosomes (approximately 150-300bp), di-nucleosomes (approximately 300-475bp), and poly-nucleosome (> 475bp). Multi-mapping and mitochondrial reads were excluded.

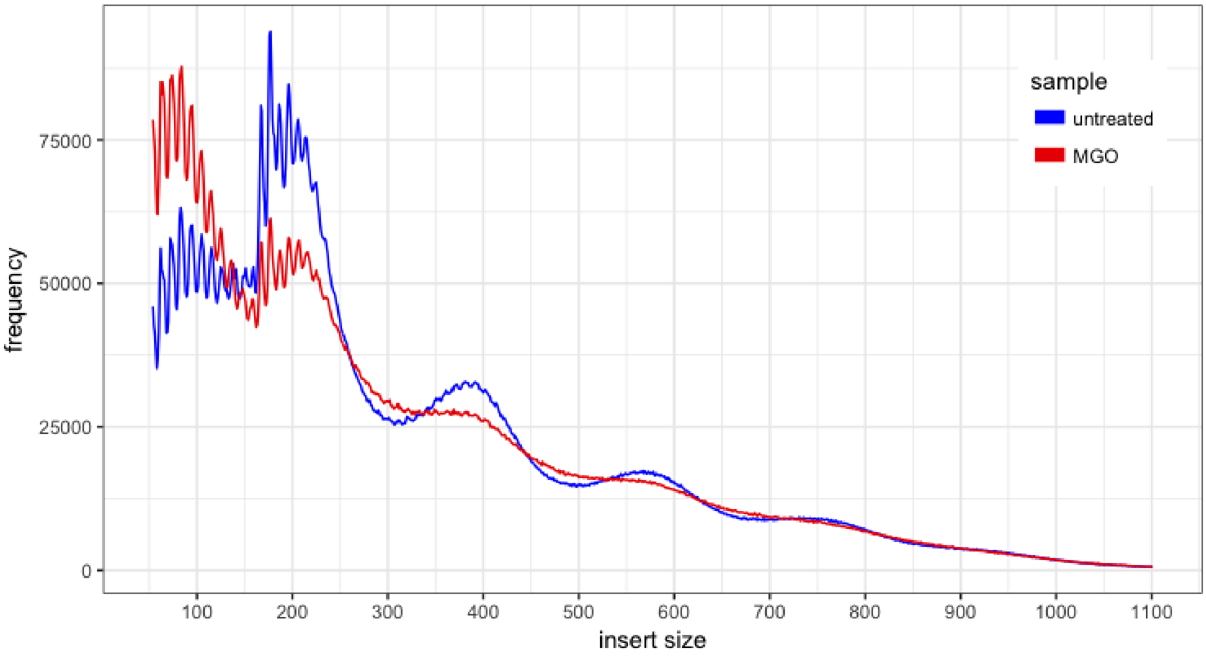

Supplementary Figure 5. Biotinylated H3 peptide synthesis. H3 peptide corresponding to amino acids 1-18 was synthesized with a C-terminal biotinylated lysine by fmoc SPPS as described in materials and methods. Peptide purification was evaluated by RP-HPLC C18 and accurate mass detected on a HPLC-ESI-MS.

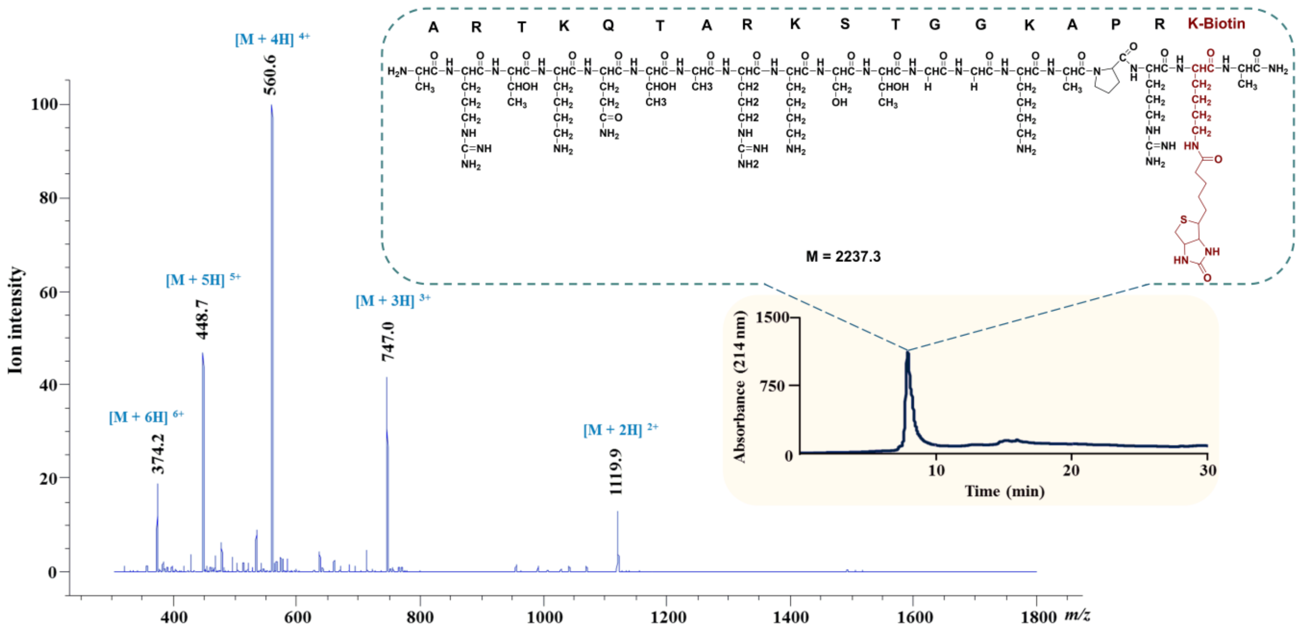

Supplementary Figure 6. Recombinant expression and purification of wild type and C106A mutant DJ-1. His_6 tagged constructs were expressed in *E. coli* Rosetta (DE3) as described in materials and methods. Briefly, the bacterial cells were lysed by sonication, cleared by centrifugation and loaded on Ni-NTA agarose column. Bound protein was washed and eluted with imidazole followed by desalting. Purified protein was loaded on SDS-PAGE and analyzed by coomassie brilliant blue stain.

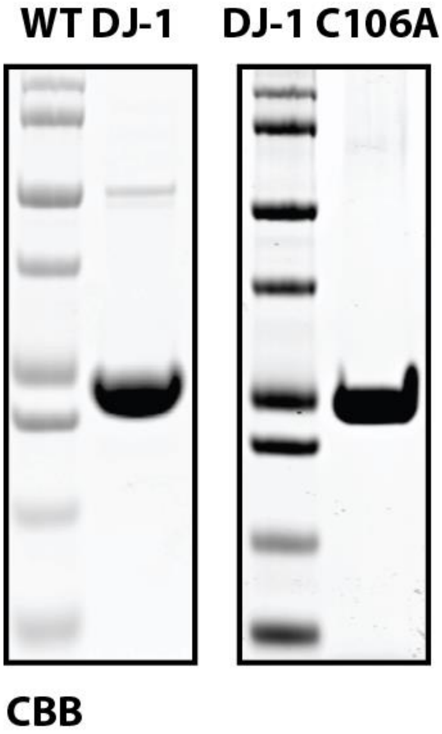

Supplementary Figure 7. NCPs glycation and deglycation. Samples from figure 4c were analyzed on a native gel followed by a western blot analysis with the indicated antibodies.

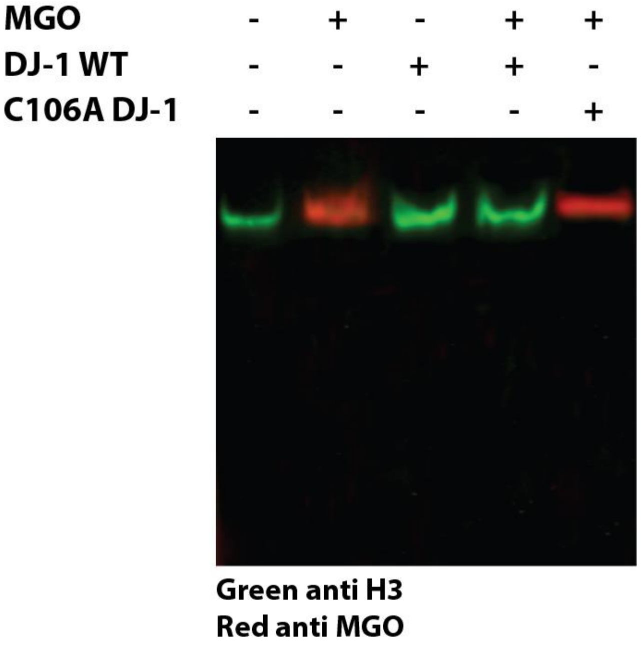

Supplementary Figure 8. Overexpression of DJ-1 in MGO treated 293T cells. Samples from Figure 4d were analyzed for DJ-1 content. 293T cells were transfected with Myc-tagged DJ-1 (either wild type or C106A) and 24 hours later were treated with 0.5 mM MGO for 12 hours. Cells were then harvested and histones were extracted with high salt and analyzed by western blot with anti DJ-1, anti Myc and anti actin as loading control.

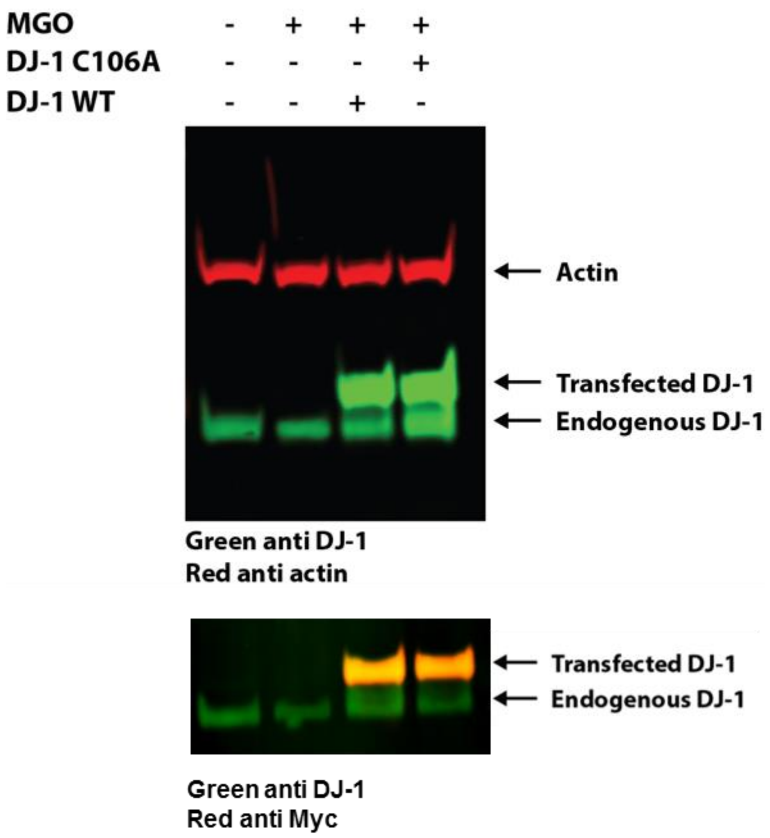

Supplementary Figure 9. Higher DJ-1 expression level is correlated with better survival in in the presence of high MGO concentration. Cells were either untreated, transfected to overexpress or downregulate DJ-1 for 24 hours. Next, cells were treated with increasing concentrations of MGO for 12 hours after which it was subjected to a viability assay as described in Materials and Methods. The error bars represent the standard deviation from three different experiments.

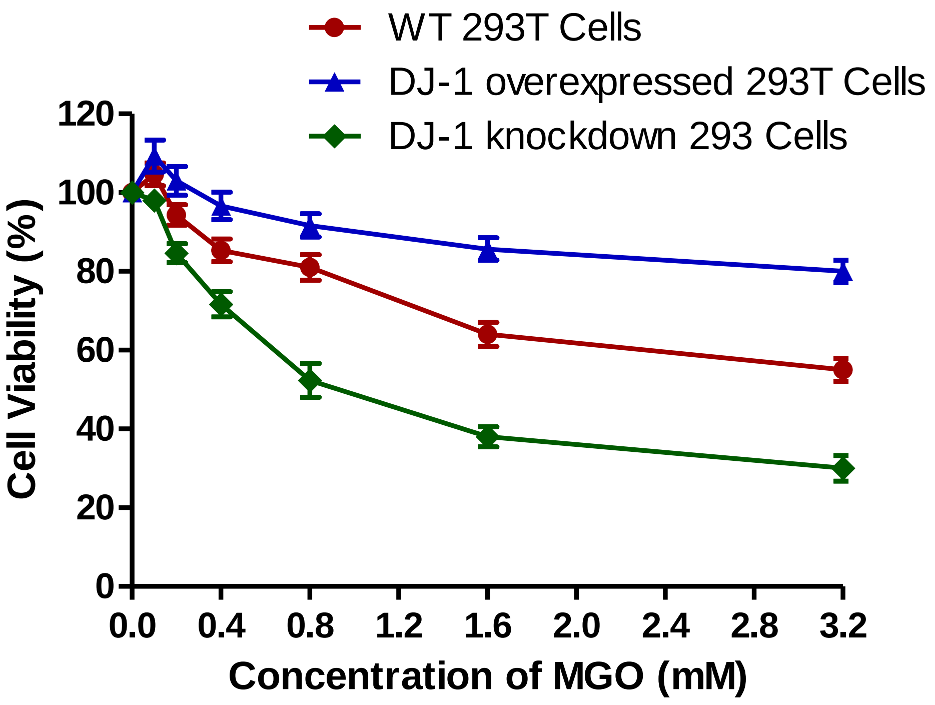

Supplementary Figure 10. DJ-1 expression levels affect histone PTMs levels. 293T cells were transfected with wild type, C106A mutant or shDJ-1 construct for 24 hours and then either untreated or treated with 0.5 mM MGO for 12 hours. Histones were extracted and analyzed by western blot with the indicated antibodies illustrating that overexpression of wild type (but not C106A mutant) DJ-1 can rescue histone PTM levels perturbed by MGO or shDJ-1.

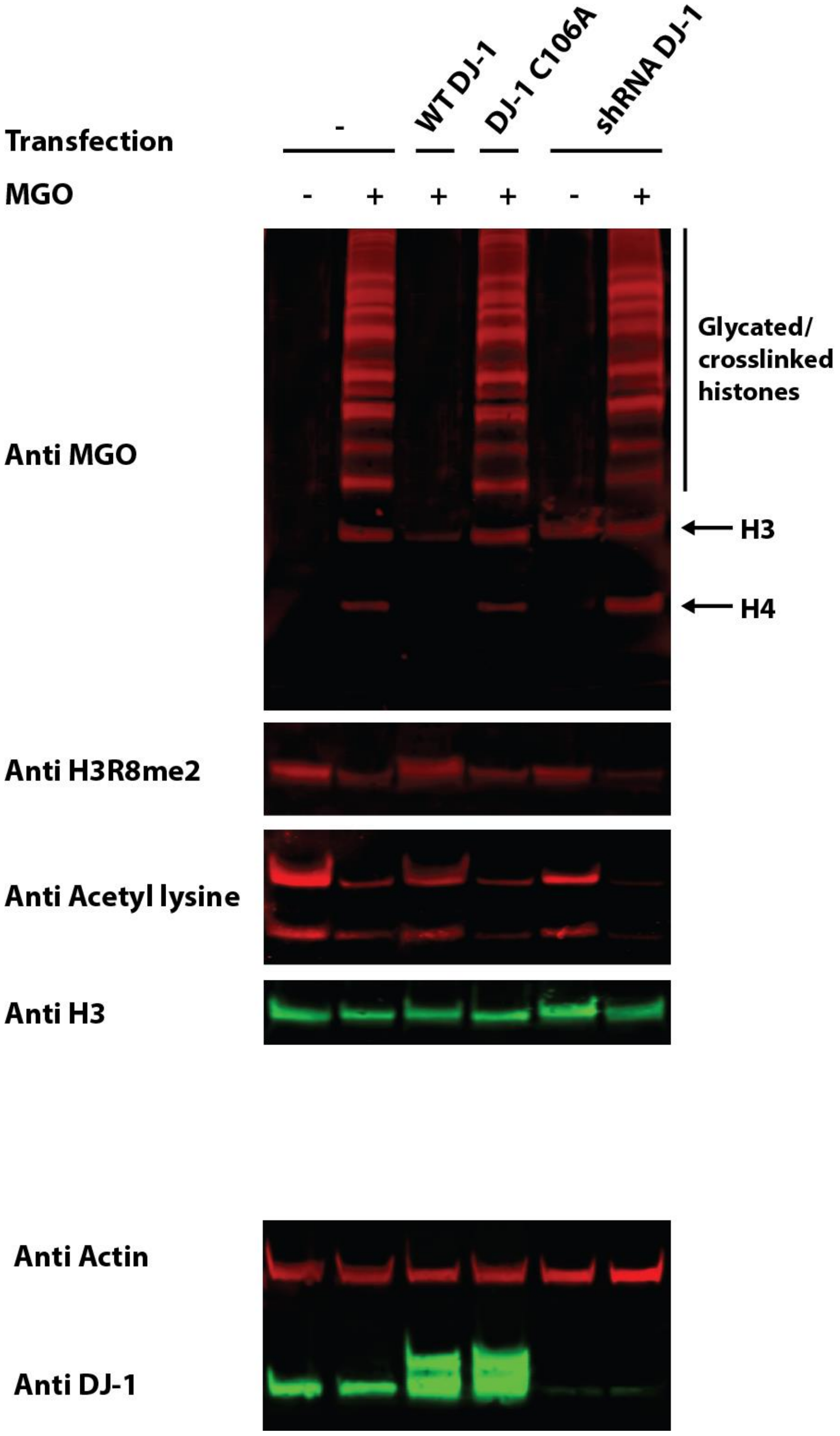

Supplementary Figure 11. DJ-1 expression in breast cancer cell lines and tumors. (a) Control (293T) and indicated breast cancer cells were fractionated, and the nuclear fraction was analyzed by western blot for DJ-1 presence and MEK ½ nuclear marker. (b) Indicated breast cancer mouse Xenografts tumors were homogenized, lysed and soluble fraction was analyzed by western blot with anti MGO, anti DJ-1 and anti actin for equal loading. (c) Indicated breast cancer tumors (T) and adjacent normal tissue (NT) from five different patients (P1-P5) were homogenized, lysed and soluble fraction was analyzed by western blot with anti DJ-1 and anti actin for equal loading.

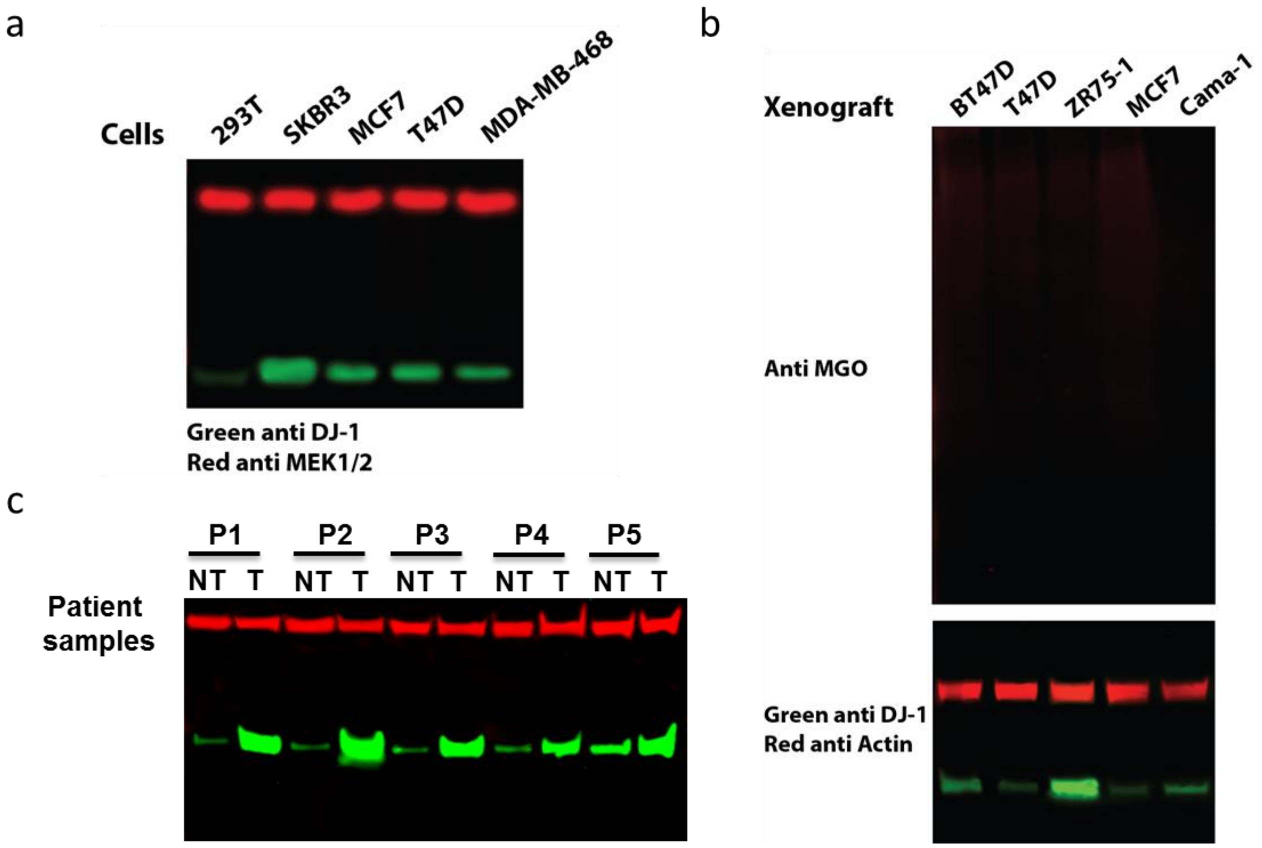

Supplementary Figure 12. SKBR3 show lower viability after DJ-1 Knockdown. SKBR3 cells were transfected with shDJ-1 for 24 hours after which they were subjected to a viability test in the absence of MGO. (a) Viability test showing relative lower survival to knockdown cells. The error bars represent the standard deviation from three different experiments. (b) Westernblot analysis showing DJ-1 knockdown and increased basal glycation.

a

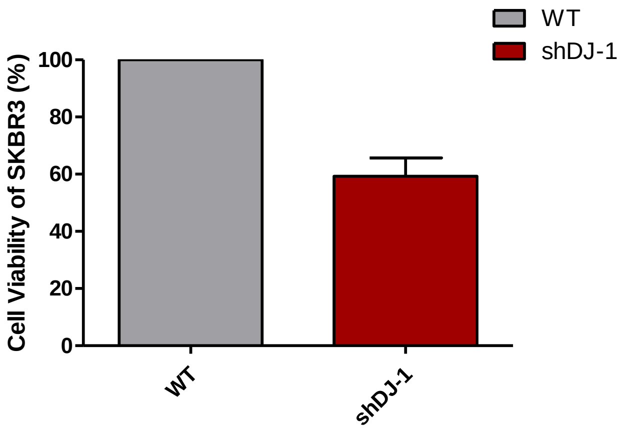

b

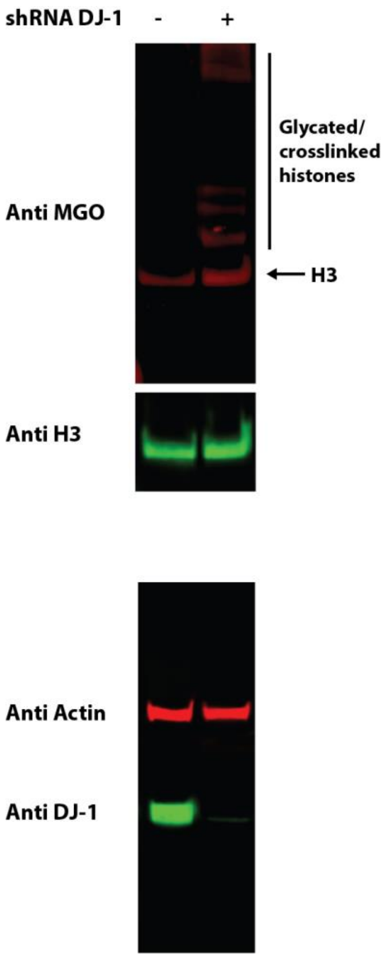

Supplementary Figure 13. Proposed mechanism of DJ-1-mediated histone deglycation. MGO can rapidly react with the primary amine groups in amino acid side chains of histones in a non-enzymatic manner. The resulting early stage adducts then rearrange into the α-hydroxypropionamide structure of which the process can be catalyzed by DJ-1. The key catalytic residue C106 in DJ-1 nucleophilicly attacks the amide bond, recovers the histone amine groups and leaves as the thioester. After the spontaneous hydrolysis, the MGO is finally converted into lactate species.

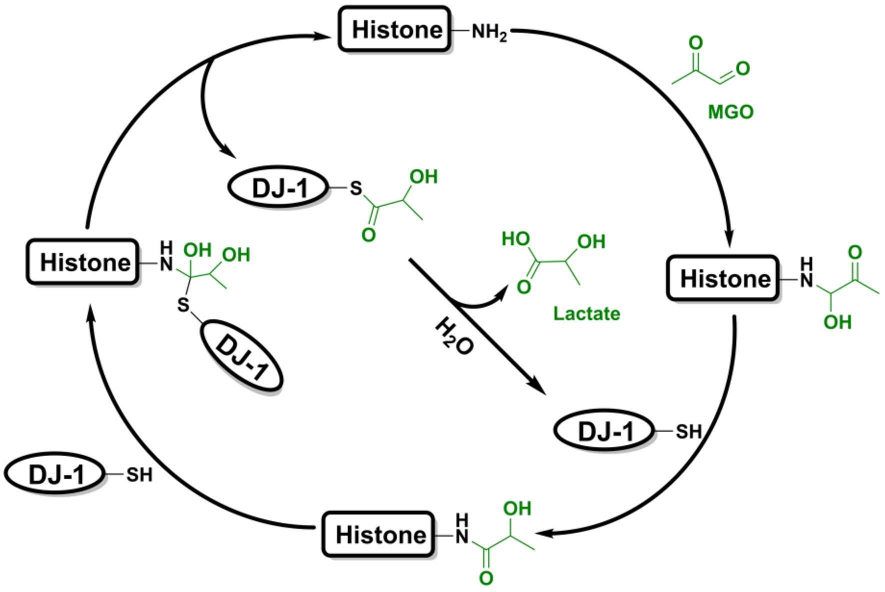

Supplementary Figure 14. Full gels and uncropped immunoblots.

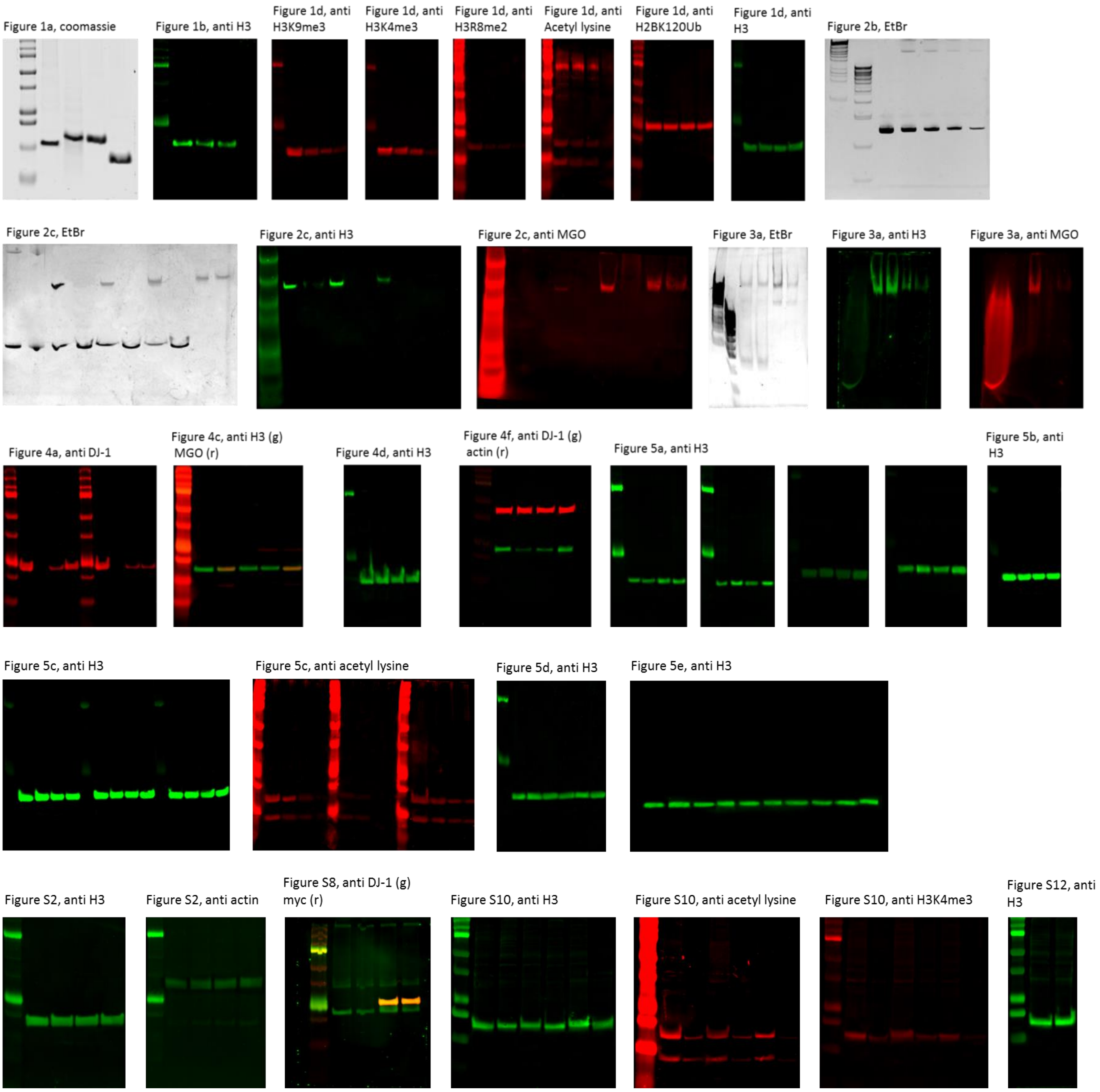

Supplementary Table 1. Primary antibodies used in this manuscript.

Host	Epitope	WB	Vendor
Mouse	Anti-MGO	1: 500	Cell Biolabs (STA-011)
Chicken	Anti-H3	1: 1000	Abcam (ab134198)
Mouse	Anti-H3	1: 1000	Abcam (ab10799)
Rabbit	Anti-DJ-1	1: 1000	CST (2134S)
Mouse	Anti-Myc	1:1000	Abcam (ab18185)
Mouse	Anti-Actin	1: 1000	CST (3700S)
Mouse	Anti-MEK 1/2	1: 1000	CST (4694S)
Rabbit	Anti-Biotin	1: 1000	Abcam (ab53494)
Rabbit	Anti-H3K4Me3	1: 1000	CST (9751S)
Rabbit	Anti-H3K9Me3	1: 1000	CST (13969S)
Rabbit	Anti-Acetyl Lys	1: 1000	CST (9441S)
Rabbit	Anti-H2BK120Ub	1: 1000	CST (5546S)

Supplementary Table 2. Secondary antibodies used in this manuscript.

Host	Epitope	Label	Dilution	Vendor
Donkey	Anti-Chicken	IRDye 800CW	1: 15000	Li-Cor
Goat	Anti-Mouse	IRDye 680RD	1: 15000	Li-Cor
Goat	Anti-Mouse	IRDye 800CW	1: 15000	Li-Cor
Goat	Anti-Rabbit	IRDye 800CW	1: 15000	Li-Cor
Goat	Anti-Rabbit	IRDye 680RD	1: 15000	Li-Cor

Supplementary Table 3. MGO treatments used in this study.

Substrate	Experiment	Methylglyoxal concentration	Stoichiometry of sites/MGO	Reaction time
Free histones	*in vitro*	0.1 mM	4:1	12 hr
		0.5 mM	1:1	
		1.0 mM	1:2	
Nucleosomes	*in vitro*	0.5mM	1:2	72 hr
		2.0 mM	1:8	
		5.0 mM	1:20	
		30 mM	1:120	
		100 mM	1:400	
12-mer arrays	*in vitro*	1 mM	1:0.3	≤ 24 hr
		2 mM	1:0.6	(6, 12, 18 or
		10 mM	1:3	24 hr)
		100 mM	1:30	
Cultured cells	*in cellulo*	0.25 mM	N/A	12 hr
		0.5 mM		(or 24 hr)
		1.0 mM		
