# Supplementary material for: Reversible histone glycation is associated with disease-related changes in chromatin architecture
Source: Nat Commun. 2019 Mar 20;10:1289. doi: 10.1038/s41467-019-09192-z (PMC6426841; doi:10.1038/s41467-019-09192-z)
Supplement: Supplementary file 2 — Reporting Summary [file 41467_2019_9192_MOESM2_ESM.pdf]

nature research

Corresponding author(s): Yael David

Reporting Summary

Nature Research wishes to improve the reproducibility of the work that we publish. This form provides structure for consistency and transparency in reporting. For further information on Nature Research policies, see Authors & Referees and the Editorial Policy Checklist.

Statistical parameters

When statistical analyses are reported, confirm that the following items are present in the relevant location (e.g. figure legend, table legend, main text, or Methods section).

n/a	Confirmed	
☐	☒	The exact sample size (n) for each experimental group/condition, given as a discrete number and unit of measurement
☐	☒	An indication of whether measurements were taken from distinct samples or whether the same sample was measured repeatedly
☒	☐	The statistical test(s) used AND whether they are one- or two-sided<br>*Only common tests should be described solely by name; describe more complex techniques in the Methods section.*
☒	☐	A description of all covariates tested
☒	☐	A description of any assumptions or corrections, such as tests of normality and adjustment for multiple comparisons
☐	☒	A full description of the statistics including central tendency (e.g. means) or other basic estimates (e.g. regression coefficient) AND variation (e.g. standard deviation) or associated estimates of uncertainty (e.g. confidence intervals)
☒	☐	For null hypothesis testing, the test statistic (e.g. F, t, r) with confidence intervals, effect sizes, degrees of freedom and P value noted<br>*Give P values as exact values whenever suitable.*
☒	☐	For Bayesian analysis, information on the choice of priors and Markov chain Monte Carlo settings
☒	☐	For hierarchical and complex designs, identification of the appropriate level for tests and full reporting of outcomes
☒	☐	Estimates of effect sizes (e.g. Cohen's d, Pearson's r), indicating how they were calculated
☐	☒	Clearly defined error bars<br>*State explicitly what error bars represent (e.g. SD, SE, CI)*

Our web collection on statistics for biologists may be useful.

Software and code

Policy information about availability of computer code

Data collection	No special software was used for data collection.
Data analysis	OriginPro v2017, GraphPad Prism 5, Python (3.4.2), R (3.4.3) on RStudio (1.0.143), ggplot2 (2.2.1), BWA-MEM (0.7.15), Picard Tools (2.18.4), MACS2 (2.1.0) run on Python (2.7.0), IDR (2.0.2; https://github.com/nboley/idr), NucleoATAC (0.2.1) run on Python (2.7.0), deepTools (3.0.2)

For manuscripts utilizing custom algorithms or software that are central to the research but not yet described in published literature, software must be made available to editors/reviewers upon request. We strongly encourage code deposition in a community repository (e.g. GitHub). See the Nature Research guidelines for submitting code & software for further information.

Data

Policy information about availability of data

All manuscripts must include a data availability statement. This statement should provide the following information, where applicable:

- Accession codes, unique identifiers, or web links for publicly available datasets
- A list of figures that have associated raw data
- A description of any restrictions on data availability

Raw and processed sequencing data are available from the Gene Expression Omnibus (GEO) database under accession code GSE121252. All the other data are available from the corresponding author upon request.

Field-specific reporting

Please select the best fit for your research. If you are not sure, read the appropriate sections before making your selection.

☒ Life sciences ☐ Behavioural & social sciences ☐ Ecological, evolutionary & environmental sciences

For a reference copy of the document with all sections, see nature.com/authors/policies/ReportingSummary-flat.pdf

Life sciences study design

All studies must disclose on these points even when the disclosure is negative.

Sample size	No statistical methods were used to pre-determine sample size. Sample sizes were chosen as n ≥ 3.
Data exclusions	No data was excluded from the analysis.
Replication	All the data in this paper have been repeated for more than 3 times.
Randomization	Animals were randomized according to weight at the start of studies.
Blinding	Blinding was not necessary as we were observing large differences in staining or morphology.

Reporting for specific materials, systems and methods

Materials & experimental systems

n/a	Involved in the study	
☒	☐	Unique biological materials
☐	☒	Antibodies
☐	☒	Eukaryotic cell lines
☒	☐	Palaeontology
☐	☒	Animals and other organisms
☒	☐	Human research participants

Methods

n/a	Involved in the study	
☒	☐	ChIP-seq
☒	☐	Flow cytometry
☒	☐	MRI-based neuroimaging

Antibodies

Antibodies used	Mouse Anti-Methylglyoxal Monoclonal Antibody (Cell Biolabs, STA-011), Chicken Anti-H3 Polyclonal Antibody (Abcam, ab134198), Mouse Anti-H3 Monoclonal Antibody (Abcam, ab10799), Rabbit Anti-DJ-1 Monoclonal Antibody (CST, 2134S), Mouse Anti-Myc tag Monoclonal Antibody (Abcam, ab18185), Mouse Anti-Actin Monoclonal Antibody (CST, 3700S), Mouse Anti-MEK1/2 Monoclonal Antibody (CST, 4694S), Rabbit Anti-Biotin Polyclonal Antibody (Abcam, ab53494), Rabbit Anti-H3K4Me3 Monoclonal Antibody (CST, 9751S), Rabbit Anti-H3K9Me3 Monoclonal Antibody (CST, 13969S), Rabbit Anti-H3R8Me2 Monoclonal Antibody (CST, 13939S), Rabbit Anti-Acetyl Lys Monoclonal Antibody (CST,), Rabbit Anti-H2BK120Ub Monoclonal Antibody (CST, 5546S)
Validation	Each primary antibody used in this study was validated for species (human or mouse) and application (immunblotting and IP) by the above-listed manufacturer using recombinant protein and/or expressing cell types as a positive control; these validation studies are reported for each primary antibody on the product website. In addition, primary antibodies were validated for species and application (immunblotting and IP) in our experiments using recombinant proteins and/or expressing cell types as a positive control and are reported in main text and supplemental figures.

Eukaryotic cell lines

Policy information about cell lines

Cell line source(s)	HEK293T, SKBR3, MCF7, T47D, MDA-MB-468, BT47D, T47D, ZR75-1, MCF7 and Cama-1 cell lines used in this study were all sourced from ATCC.
Authentication	All cell lines used in this manuscript were authenticated by the vendors where we acquired these cell lines. ATCC used morphology, kryotyping, and PCR-based approaches.
Mycoplasma contamination	All cell lines were tested for mycoplasma monthly (MycoFluor Mycoplasma detection kit, Invitrogen) and tested negative for contamination.
Commonly misidentified lines (See ICLAC register)	No commonly misidentified cell lines were used in this study.

Animals and other organisms

Policy information about studies involving animals; ARRIVE guidelines recommended for reporting animal research

Laboratory animals	Six-to-8-week-old nu/nu athymic BALB/c female mice (Harlan Laboratories, Inc.) were used for the breast cancer xenografts experiments.
Wild animals	The study did not involve any wild animals.
Field-collected samples	The study did not involve any samples collected from the field.
